# c-Abl Inhibitors Enable Insights into the Pathophysiology and Neuroprotection in Parkinson’s Disease

**DOI:** 10.3389/fnagi.2016.00254

**Published:** 2016-10-26

**Authors:** Dan Lindholm, Dan D. Pham, Annunziata Cascone, Ove Eriksson, Krister Wennerberg, Mart Saarma

**Affiliations:** ^1^Medicum, Department of Biochemistry and Developmental Biology, Faculty of Medicine, University of HelsinkiHelsinki, Finland; ^2^Minerva Foundation Institute for Medical Research, Biomedicum Helsinki 2U, HelsinkiFinland; ^3^Institute for Molecular Medicine Finland, University of HelsinkiHelsinki, Finland; ^4^Institute of Biotechnology, University of HelsinkiHelsinki, Finland

**Keywords:** Parkinson’s disease, α-synuclein, parkin, c-Abl, nilotinib, leukemia

## Abstract

Parkinson’s disease (PD) is a progressive neurodegenerative disorder causing movement disabilities and several non-motor symptoms in afflicted patients. Recent studies in animal models of PD and analyses of brain specimen from PD patients revealed an increase in the level and activity of the non-receptor tyrosine kinase Abelson (c-Abl) in dopaminergic neurons with phosphorylation of protein substrates, such as α-synuclein and the E3 ubiquitin ligase, Parkin. Most significantly inhibition of c-Abl kinase activity by small molecular compounds used in the clinic to treat human leukemia have shown promising neuroprotective effects in cell and animal models of PD. This has raised hope that similar beneficial outcome may also be observed in the treatment of PD patients by using c-Abl inhibitors. Here we highlight the background for the current optimism, reviewing c-Abl and its relationship to pathophysiological pathways prevailing in PD, as well as discussing issues related to the pharmacology and safety of current c-Abl inhibitors. Clearly more rigorously controlled and well-designed trials are needed before the c-Abl inhibitors can be used in the neuroclinic to possibly benefit an increasing number of PD patients.

Age is a major risk factor for Parkinson’s disease (PD), but the precise molecular mechanisms underlying the disease are not fully understood. Recent advances in genetics and pathophysiology of PD have increased our understanding about the fundamental processes contributing to disease pathogenesis but in the majority of cases the precise etiology is unknown. However, evidence suggests that protein aggregation, mitochondrial dysfunctions, ER stress, neuroinflammation and reduced growth factor levels contribute to neurodegeneration and play key roles in PD (Gupta et al., 2008; Fahn, 2010; Mullin and Schapira, 2015). Studies of gene mutations causing familial PD have identified proteins such as α-synuclein and the E3 ubiquitin ligase, Parkin that are important also in the more common sporadic forms of PD (Singleton et al., 2003; Dawson and Dawson, 2011). One hallmark of PD is the aggregation of α-synuclein in the complex with other proteins in so called Lewy bodies particularly within dopaminergic neurons in the midbrain, though the precise role of this protein accumulation is currently unclear (Spillantini et al., 1997; Winslow and Rubinsztein, 2011). Defects in protein handling involving dysfunctional autophagy, and the ubiquitin-proteasome system as well as ER stress and changes in posttranslational modifications of proteins with altered cell signaling cascades can contribute to the process of protein accumulation and degeneration of dopamine neurons (Lindholm et al., 2006; Winslow and Rubinsztein, 2011; Klionsky et al., 2016).

In PD, the levels of the neurotransmitter dopamine decrease in the brain caused by the degeneration and final loss of dopamine neurons in substantia nigra. Many symptoms especially those related to altered movement patterns in PD can be treated with L-Dopa, a drug restoring the levels of dopamine. However, L-Dopa does not halt the progressive decline in dopamine neurons, and may in the long term produce severe dyskinesia. It is thus of utmost importance to find therapies to counteract the neurodegeneration and restore the neuronal circuits in the brain of PD patients. Previous research on pathophysiological processes in PD has also helped to identify novel and promising targets for neuroprotection (Brundin et al., 2015; Kalia et al., 2015; Lindholm et al., 2016). Unfortunately many novel molecules and trophic factors showing promising data in preclinical experiments and animal models of PD have failed to recapitulate these in more rigorous clinical trials (Kalia et al., 2015; Lindholm et al., 2016).

It is therefore gratifying to note that older drugs previously used for treatment of other disorders may have potentially beneficial effects in neurodegenerative diseases including PD (Patrone et al., 2014). Among these are the thiazolidine drugs used in the treatment of type-2 diabetes that act via the nuclear peroxisome proliferator-activated receptor-γ co-activator -1α (PGC-1α), as well as the more novel compounds like exenatide stimulating glucagon-like peptide receptor (Aviles-Olmos et al., 2014; Patrone et al., 2014). Preclinical studies of these compounds have revealed significant neuroprotective effects in animal models of PD and in other brain disorders. The recent additions to this list of promising disease-modifying compounds in PD include small molecule inhibitors targeting the activity of the c-Abl tyrosine kinase (Hebron et al., 2013; Imam et al., 2013; Karuppagounder et al., 2014; Tanabe et al., 2014).

## What is c-Abl?

**Figure 1:**
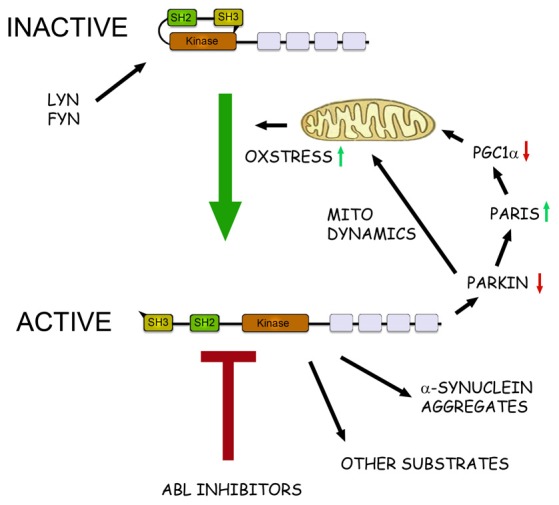
**The role of c-Abl in pathophysiological events in Parkinson’s disease (PD).** Schematic view of the structure and action of c-Abl in neurons. c-Abl contains the protein kinase region and the SH2 and SH3 domains. The activity of the protein is tightly regulated in cells by intramolecular and other interactions and by the Src family kinases Lyn and Fyn. In addition, increased oxidative stress involving mitochondria can activate c-Abl leading to subsequent phosphorylation of downstream targets. In the neurons α-synuclein form intracellular aggregates called Lewy bodies and this process is aggravated after phosphorylation by c-Abl. Parkin is an ubiquitin ligase that regulates the mitochondrial biogenesis via the PARkin Interacting Substrate (Paris) and the transcriptional coactivator Peroxisome proliferator-activated receptor-γ coactivator-1α (PGC1α) Parkin also controls mitochondrial dynamics and mitophagy. Phosphorylation by c-Abl inactivates Parkin leading to mitochondrial alterations and an increased oxidative stress. This may cause further activation of c-Abl producing a vicious cycle in the neuron with an enhanced cell degeneration to follow. c-Abl inhibitors may potentially hinder this cascade by reducing the amount of α-synuclein aggregates and by restoring the functions of Parkin and of other substrates. Some of the c-Abl inhibitors are known to block also the Src family kinases and may thus theoretically be more neuroprotective. A challenge for the c-Abl inhibitors is that these drugs may not effectively pass the blood-brain barrier (BBB) possibly limiting their use in PD and other brain disorders.

c-Abl (ABL1) is the cellular homolog of the Abelson murine leukemia virus oncogene and belongs to the Abl family of tyrosine kinase present in the cytoplasm and nucleus of the cell. c-Abl is expressed in most cells and is part of an intricate network of protein interactions and phosphorylation events in the cell (Hantschel and Superti-Furga, 2004). Thus c-Abl is involved in a variety of physiological functions including the regulation of cell growth and motility, cytoskeleton dynamics, receptor endocytosis, DNA repair, cell survival and autophagy (Hantschel and Superti-Furga, 2004). c-Abl is normally present in an inactive form in the cell and its activity is tightly regulated by intramolecular bonds, as well as by binding to protein complexes, and linkage to membranes via an aminoterminal myristoyl group c-Abl is activated following auto-phosphorylation and by the action of other kinases including Lyn and Fyn that are Src-family kinases (Figure 1). c-Abl is also activated by DNA damage and during cell stress involving an increased production of reactive oxidative species that ultimately can cause cell degeneration (Hantschel and Superti-Furga, 2004). c-Abl influences several cell processes acting in concert with other kinases and the key protein targets of c-Abl may vary in different cells. The consensus sequence for target phosphorylation by c-Abl is also rather flexible (see, www.kinasenet.ca), so that novel protein targets for the kinase should be experimentally verified under actual physiological conditions.

c-Abl has been previously studied in the context of human cancers, and mutations in the protein can lead to an enhanced protein kinase activity with an increased cell proliferation (Hantschel and Superti-Furga, 2004). In chronic myeloid leukemia (CML) there is a chimeric Breakpoint cluster region protein (BCR)-ABL1 fusion gene present in cells caused by the translocation between chromosomes 22 (BCR) and 9 (ABL1) (9;22; q34;q11) producing a fusion gene, encoding a highly active kinase (Hantschel and Superti-Furga, 2004). The BCR-ABL1 fusion is part of the criteria of the CML diagnosis, but it is also found in about one-third of adult patients with acute lymphoblastic leukemia (ALL) and in about 10% ALL children. It is thought that the differentiation of myeloid precursor cells is particularly inhibited by the activity of BCR-ABL1 protein, but other cellular processes are also involved (Quintás-Cardama and Cortes, 2009).

In contrast to tumor cells, the activity of c-Abl in post-mitotic neurons is linked to the regulation of the cytoskeleton, to neurite outgrowth, to neuronal plasticity and to the control of cell stress (Schlatterer et al., 2011). Data from animal models have shown that c-Abl is activated in several brain disorders characterized by an increased oxidative stress, including PD (Schlatterer et al., 2011). c-Abl was also found to be activated (more phosphorylated) in brain specimen from PD patients suggesting a pathophysiological role of c-Abl in the disease (Ko et al., 2010; Imam et al., 2011; Brahmachari et al., 2016). In support of this, conditional gene deletion of *Abl* in the mouse brain protected dopaminergic neurons in the N-methyl-4-phenyl-1,2,3,6-tetrahydroyridine (MPTP) neurotoxin model of PD (Ko et al., 2010). In addition, tyrosine kinase inhibitors against c-Abl are neuroprotective and can modulate phosphorylation of specific protein targets in the brain (see below).

## Inhibitors Against c-Abl

Given the importance of BCR-ABL in tumors, specific inhibitors against the Abl kinase have been searched for the treatment of blood cancer. Imatinib (STI571, Gleevec/Glivec) was the first c-Abl tyrosine kinase inhibitor identified and has been used in the treatment of CML now for several years (Capdeville et al., 2002; Heffron, 2016). While imatinib generally is well tolerated, resistance occur in a portion of CML patients over time, and indicative of the essential driver role of BCR-ABL1 in CML. Resistance is most commonly mediated by mutations in the BCR-ABL1 kinase domain that render the kinase insensitive to imatinib. For this reason, c-Abl inhibitors that are also active against the most common imatinib resistance mutations have been designed that can be used in cases when imatinib resistance occur (Musumeci et al., 2012; Heffron, 2016).

A list of selected c-Abl inhibitors is presented in Table 1 (Heffron, 2016). The presence of a blood-brain barrier (BBB) effectively hinders most drugs to reach the brain and there are also active efflux mechanisms further reducing the drug concentration in the tissue (Heffron, 2016). For treatment of brain tumors in leukemia, dasatinib (Das et al., 2006) was shown to penetrate the BBB (Porkka et al., 2008), whereas imanitib and ponatinib (Huang et al., 2010) do so to a limited extent (Abid and De Mel, 2016; Heffron, 2016). Regarding nilotinib and bosutinib (Remsing Rix et al., 2009) recent studies in animal models of Alzheimer’s disease have shown that these drugs show a partial BBB penetrance (Lonskaya et al., 2015). It should be remembered that the state of the BBB may change in animal models of brain disease not reflecting the real situation in human patients. Moreover, the optimal concentrations of drug required to treat neurodegenerative diseases are largely unknown.

**Table 1:** **Some properties of current c-Abl inhibitors**.

Compound	IC50 for c-Abl (nM) in cells	Dosage (mg/day)	Brain penetration	Safety tolerability
Imatinib (Gleevec/Glivec)	100-200	400-600	Poor	Good
Nilotinib (Tasigna)	5-10	400-600	Moderate	Good*
Dasatinib (Sprycel)	0.25-0.5	100-180	Moderate	Good
Bosutinib (Bosulif)	10-20	500-600	Moderate	Good
Ponatinib (Iclusig)	0.25-0.5	15-45	Limited	Moderate**
Radotinib*** (Supect)	20-40	600-800	Poor	Good

*Black label warning for possible cardiac side effects. **Black label warning for possible cardiac or liver side effects. ***Only approved in South Korea.

Abl inhibitor therapy in CML is typically handled as a chronic treatment with patients receiving the drugs for years to decades. The Abl inhibitors do therefore have to be well tolerated (Pinilla-Ibarz et al., 2015), arguing for that they could also be used in continuous treatment of PD with the caution that different dosing could be needed, hence causing new adverse effects. Intermittent dosing of Abl inhibitors works well for treatment of CML (Shah et al., 2008) but it is possible that more constant serum concentrations will be needed to produce an effect in neurodegenerative disorders. Issues related to pharmacodynamics and safety profile of the c-Abl inhibitors in neurodegenerative treatment deserve particular attention before a clinical use of the drugs in PD can be considered.

## Nilotinib and PD

Nilotinib (AMN107, Tasigna) is a second-generation c-Abl tyrosine kinase inhibitor that is used in CML in a daily dose of 300 mg to over 1000 mg per patient (Weisberg et al., 2006). The drug is relatively safe and tolerated but in some patients heart complications have been associated with the use of nilotinib, and therefore a black box warning has been given to the drug by the USA Food and Drug Administration.

As discussed above, preclinical studies show that nilotinib penetrates into brain tissue and was neuroprotective in two animal models of PD; the MPTP neurotoxin model, and the one induced by lentiviral overexpression of α-synuclein in the midbrain (Hebron et al., 2013; Karuppagounder et al., 2014; Tanabe et al., 2014). Notably the drug improved motor behavior in these mice, consistent with a beneficial effect on neuronal circuity involved in movement control. Nilotinib also increased concentrations of dopamine and its metabolites, homovalinic acid and 3,4-dihydroxyphenylacetic acid, in brains of MPTP treated animals (Hebron et al., 2013; Karuppagounder et al., 2014). It remains to be studied whether nilotinib may also have restorative and regenerative functions for dopaminergic neurons and using animal models that resemble more closely the progressive nature and ageing aspects of PD in human patients.

A recent clinical study showed that nilotinib reduced the relative level of phosphorylated (active) c-Abl in the cerebrospinal fluid of treated PD patients (Pagan et al., 2016).

In search for biochemical correlates for the cytoprotective effect of nilotinb in brain, mainly two proteins have been in focus (see Figure 1). Parkin is mutated in autosomal recessive parkinsonism but it also plays a role in the more common sporadic PD by affecting ubiquitination of protein targets (Dawson and Dawson, 2011). Among substrates for Parkin the protein, PARkin Interacting Substrate (Paris) inhibits the nuclear coactivator PGC-1α that in turn regulates mitochondrial biogenesis and functions (Shin et al., 2011). Inactivation of, or mutations in Parkin increases levels of Paris and reduce PGC-1α that may lead to mitochondrial dysfunctions and ultimately to neuronal loss (Shin et al., 2011). In contrast expression of Parkin or PGC-1α can restore these functions and counteract the neurodegeneration (Shin et al., 2011; Mudò et al., 2012). It was subsequently shown that c-Abl phosphorylates Parkin reducing its neuroprotective ability, whilst treatment with nilotinib increased Parkin activity (Ko et al., 2010; Dawson and Dawson, 2011; Imam et al., 2011). In these studies, postmortem brain samples from PD patients showed an increase in c-Abl activity along with higher level of Parkin phosphorylation.

In PD, α-synuclein aggregates to form insoluble fibrils in pathological conditions characterized by Lewy bodies. α-synuclein is degraded mainly via autophagy including chaperon-mediated autophagy (Cuervo et al., 2004), and overexpression of and modifications/mutations in α-synuclein in turn, can influence autophagy (Winslow and Rubinsztein, 2011; Klionsky et al., 2016). Recently it was shown that α-synuclein is phosphorylated by c-Abl at the tyrosine residue 39 that negatively regulates its clearance from the cell (Hebron et al., 2013; Mahul-Mellier et al., 2014; Brahmachari et al., 2016). In contrast, nilotinib enhanced α-synuclein degradation in cultured cells and in animal models of PD. Along with this, *c-Abl* gene deleted mice have a reduced α-synuclein aggregation and toxicity (Brahmachari et al., 2016). The link between c-Abl and α-synuclein seems reciprocal as the overexpression of α-synuclein can increase c-Abl activity and thereby compromise autophagy (Hebron et al., 2013).

In Figure 1 we schematically summarize the actions of c-Abl and its inhibition in the regulation of cell viability in dopaminergic neurons and the involvement of the protein targets, α-synuclein and Parkin (Figure 1). In nilotinib-treated neurons, Parkin and α-synuclein may reinforce each others actions thereby contributing to a positive effect of the drug against cell degeneration. One additional target for c-Abl deserving to be mentioned here is the cyclin-dependent kinase 5 (Cdk5) that is highly expressed in brain neurons and shown to be activated in neurodegenerative diseases including PD (Smith et al., 2003; Wen et al., 2014). Cdk5 has several targets in neurons and influences synaptic plasticity and cell death. Cdk5 is phosphorylated by glutamate during excitotoxicity (Putkonen et al., 2011) and by c-Abl during oxidative stress in animal model of PD (Yamamura et al., 2013). This suggests an important link between active c-Abl and Cdk5 in the regulation of neuronal signaling and viability that warrants further studies.

It is important to note that the actual dose of nilotinib and other c-Abl inhibitors required to achieve an optimal neuroprotection in PD is currently not known. Moreover, α-synuclein accumulates also in other forms of α-synucleinopathies (Spillantini and Goedert, 2016), and in Dementia with Lewy bodies (DLB). It remains to be studied whether and to what extent c-Abl is altered in these disorders and whether an inhibition of c-Abl may offer novel strategies for treatment.

Recently nilotinib was employed in an open-labeled trial to treat 12 patients afflicted by either PD or DLB (Pagan et al., 2016). Since the study lacked proper controls the results are preliminary and should be treated with caution. The data showed that administration of nilotinib in a daily dose of 150 mg or 300 mg, lower than the amounts used in blood cancer therapies, was rather safe and the drug was well tolerated by the patients. Regarding the clinical outcome, there was also a possible effect on motor behavior and on cognition of treated patients but the small group size and the possibility for placebo effects are confounding factors hindering definitive conclusions.

## Conclusion

In summary, the recent preclinical data on the protective role of c-Abl inhibitors in models of PD raised hopes for the application of these compounds also in the clinics (Wyse et al., 2016). The recent small open-label drug trial using nilotinib paved the way in this direction but the results obtained are so far preliminary (Pagan et al., 2016). In this regard more carefully designed and well-controlled trials with PD patients are required to arrive at a conclusion regarding the potential benefits of c-Abl inhibitors in the treatment of PD. In particular, studies of the actual doses of nilotinib and of other c-Abl inhibitors required to achieve an optimal neuroprotection in PD are warranted to get statistic significance for the effects observed.

It is to be remembered that the penetration through the blood brain barrier varies between the different c-Abl inhibitors and this may also change during the course of the disease. In this regard the potential use of other delivery routes for these drugs may be considered, such as the intranasal application of the drug that is yet to be analyzed. However, in all planned treatments, issues related to adverse reactions and side effects of the c-Abl inhibitors have to be rigorously analyzed since a long-term treatment with these drugs is foreseen in the case of neurodegenerative diseases, such as PD.

We also think that before starting phase 2 clinical trials using c-Abl inhibitors, it is important to conduct experiments on non-human primate models of PD. Further it should be studied whether the use of c-Abl inhibitors in PD may help to treat non-motor symptoms in PD, such as loss of smell, constipation, sleep disorders, depression and cognitive impairments. In PD these symptoms derive from changes in enteric and olfactory dopamine neurons and in neuronal circuits in the brain other than the dopaminergic nigrostriatal network involved in motor control. The non-motor symptoms are equally disabling for the patients and as such require more studies using different drugs and compounds.

One lesson learned from the recent study of the thiazolidine drug pioglitazione was that despite promising preclinical data no significant neuroprotection by the drug was observed in a larger cohort of PD patients (see study, NINDS Exploratory Trials in Parkinson Disease (NET-PD) FS-ZONE Investigators (2015)). Future studies will show whether nilotinib or other c-Abl inhibitors can meet the current high expectations, and hopefully reveal disease-modifying actions in PD for the benefit of an increasing number of afflicted patients.

## Author Contributions

All authors contributed to the writing of the manuscript that was finalized by DL.

## Conflict of Interest Statement

The authors declare that the research was conducted in the absence of any commercial or financial relationships that could be construed as a potential conflict of interest.

## Abbreviations

ALL: acute lymphoblastic leukemia
BBB: blood-brain barrier
BCR: Breakpoint cluster region protein
c-Abl: cellular homolog of the Abelson murine leukemia virus oncogene
Cdk5: cyclin-dependent kinase 5
CML: chronic myeloid leukemia
Fyn: Src family tyrosine kinase
Lyn: Src family tyrosine kinase
MPTP: N-methyl-4-phenyl-1, 2, 3, 6-tetrahydroyridine
Paris: PARkin Interacting Substrate
PD: Parkinson’s disease
PGC-1α: Peroxisome proliferator-activated receptor-γ coactivator-1α.

## References

[B1] Abid M. B. De Mel S. (2016). Does ponatinib cross the blood-brain barrier? Br. J. Haematol. [Epub ahead of print]. 10.1111/bjh.14222 27352067

[B2] Aviles-Olmos I. Dickson J. Kefalopoulou Z. Djamshidian A. Kahan J. Ell P.. (2014). Motor and cognitive advantages persist 12 months after exenatide exposure in Parkinson’s disease. J. Parkinsons Dis. 4, 337–344. 10.3233/JPD-140364 24662192

[B3] Brahmachari S. Ge P. Lee S. H. Kim D. Karuppagounder S. S. Kumar M.. (2016). Activation of tyrosine kinase c-Abl contributes to α-synuclein-induced neurodegeneration. J. Clin. Invest. 126, 2970–2988. 10.1172/JCI85456 27348587 PMC4966315

[B4] Brundin P. Atkin G. Lamberts J. T. (2015). Basic science breaks through: new therapeutic advances in Parkinson’s disease. Mov. Disord. 30, 1521–1527. 10.1002/mds.26332 26177603

[B5] Capdeville R. Buchdunger E. Zimmermann J. Matter A. (2002). Glivec (STI571, imatinib), a rationally developed, targeted anticancer drug. Nat. Rev. Drug Discov. 1, 493–502. 10.1038/nrd839 12120256

[B6] Cuervo A. M. Stefanis L. Fredenburg R. Lansbury P. T. Sulzer D. (2004). Impaired degradation of mutant α-synuclein by chaperone-mediated autophagy. Science 305, 1292–1295. 10.1126/science.1101738 15333840

[B7] Das J. Chen P. Norris D. Padmanabha R. Lin J. Moquin R. V.. (2006). 2-Aminothiazole as a novel kinase inhibitor template. Structure-activity relationship studies toward the discovery of N-(2-chloro-6-methylphenyl)-2-[[6-[4–(2-hydroxyethyl)-1-piperazinyl)]-2-methyl-4 pyrimidinyl]amino)]-1,3-thiazole-5-carboxamide (dasatinib, BMS- 354825) as a potent pan-Src kinase inhibitor. J. Med. Chem. 49, 6819–6832. 10.1021/jm060727j 17154512

[B8] Dawson T. M. Dawson V. L. (2011). Parkin plays a role in sporadic Parkinson’s disease. Neurodegener. Dis. 13, 69–71. 10.1159/000354307 24029689 PMC3984465

[B9] Fahn S. (2010). Parkinson’s disease: 10 years of progress, 1997–2007. Mov. Disord. 25, S2–S14. 10.1002/mds.22796 20187239

[B10] Gupta A. Dawson V. L. Dawson T. M. (2008). What causes cell death in Parkinson’s disease. Ann. Neurol. 64, S3–S15. 10.1002/ana.21573 19127586 PMC4118469

[B11] Hantschel O. Superti-Furga G. (2004). Regulation of the c-Abl and Bcr-Abl tyrosine kinases. Nat. Rev. Mol. Cell Biol. 5, 33–44. 10.1038/nrm1280 14708008

[B12] Hebron M. L. Lonskaya I. Moussa C. E. (2013). Nilotinib reverses loss of dopamine neurons and improves motor behavior via autophagic degradation of α-synuclein in Parkinson’s disease models. Hum. Mol. Genet. 22, 3315–3328. 10.1093/hmg/ddt192 23666528 PMC3723316

[B13] Heffron T. P. (2016). Small molecule kinase inhibitors for the treatment of brain cancer. J. Med. Chem. [Epub ahead of print]. 10.1021/acs.jmedchem.6b00618 27414067

[B14] Huang W.-S. Metcalf C. A. Sundaramoorthi R. Wang Y. Zou D. Thomas R. M.. (2010). Discovery of 3–[2-(imidazo[1,2-b]pyridazin-3-yl)ethynyl]-4-methyl-N-{4-[(4-methylpiperazin-1-yl)-methyl]-3-(trifluoromethyl)phenyl}benzamide (AP24534), a potent, orally active pan-inhibitor of breakpoint cluster region-Abelson (BCR-ABL) kinase including the T315I gatekeeper mutant. J. Med. Chem. 53, 4701–4719. 10.1021/jm100395q 20513156

[B15] Imam S. Z. Trickler W. Kimura S. Binienda Z. K. Paule M. G. Slikker W. Jr.. (2013). Neuroprotective efficacy of a new brain-penetrating C-Abl inhibitor in a murine Parkinson’s disease model. PLoS One 8:e65129. 10.1371/journal.pone.0065129 23741470 PMC3669292

[B16] Imam S. Z. Zhou Q. Yamamoto A. Valente A. J. Ali S. F. Bains M.. (2011). Novel regulation of parkin function through c-Abl-mediated tyrosine phosphorylation: implications for Parkinson’s disease. J. Neurosci. 31, 157–163. 10.1523/JNEUROSCI.1833-10.2011 21209200 PMC3039694

[B17] Kalia L. V. Kalia S. K. Lang A. E. (2015). Disease-modifying strategies for Parkinson’s disease. Mov. Disord. 30, 1442–1450. 10.1002/mds.26354 26208210

[B18] Karuppagounder S. S. Brahmachari S. Lee Y. Dawson V. L. Dawson T. M. Ko H. S. (2014). The c-Abl inhibitor, nilotinib, protects dopaminergic neurons in a preclinical animal model of Parkinson’s disease. Sci. Rep. 4:4874. 10.1038/srep04874 24786396 PMC4007078

[B19] Klionsky D. J. Abdelmohsen K. Abe A. Abedin M. J. Abeliovich H. Acevedo Arozena A.. (2016). Guidelines for the use and interpretation of assays for monitoring autophagy (3rd edition). Autophagy 12, 1–222. 10.1080/15548627.2015.1100356 26799652 PMC4835977

[B20] Ko H. S. Lee Y. Shin J. H. Karuppagounder S. S. Gadad B. S. Koleske A. J.. (2010). Phosphorylation by the c-Abl protein tyrosine kinase inhibits parkin’s ubiquitination and protective function. Proc. Natl. Acad. Sci. U S A 107, 16691–16696. 10.1073/pnas.1006083107 20823226 PMC2944759

[B21] Lindholm D. Mäkelä J. Di Liberto V. Mudò G. Belluardo N. Eriksson O.. (2016). Current disease modifying approaches to treat Parkinson’s disease. Cell. Mol. Life Sci. 73, 1365–1379. 10.1007/s00018-015-2101-1 26616211 PMC11108524

[B22] Lindholm D. Wootz H. Korhonen L. (2006). ER stress and neurodegenerative diseases. Cell Death Differ. 13, 385–392. 10.1038/sj.cdd.4401778 16397584

[B23] Lonskaya I. Hebron M. L. Selby S. T. Turner R. S. Moussa C. E. (2015). Nilotinib and bosutinib modulate pre-plaque alterations of blood immune markers and neuro-inflammation in Alzheimer’s disease models. Neuroscience 304, 316–327. 10.1016/j.neuroscience.2015.07.070 26235435

[B24] Mahul-Mellier A. L. Fauvet B. Gysbers A. Dikiy I. Oueslati A. Georgeon S.. (2014). c-Abl phosphorylates α-synuclein and regulates its degradation: implication for α-synuclein clearance and contribution to the pathogenesis of Parkinson’s disease. Hum. Mol. Genet. 23, 2858–2879. 10.1093/hmg/ddt674 24412932 PMC4014189

[B25] Mudò G. Mäkelä J. Di Liberto V. Tselykh T. V. Olivieri M. Piepponen P.. (2012). Transgenic expression and activation of PGC-1α protect dopaminergic neurons in the MPTP mouse model of Parkinson’s disease. Cell. Mol. Life Sci. 69, 1153–1165. 10.1007/s00018-011-0850-z 21984601 PMC11114858

[B26] Mullin S. Schapira A. (2015). The genetics of Parkinson’s disease. Br. Med. Bull. 114, 39–52. 10.1093/bmb/ldv022 25995343

[B27] Musumeci F. Schenone S. Brullo C. Botta M. (2012). An update on dual Src/Abl inhibitors. Future Med. Chem. 4, 799–822. 10.4155/fmc.12.29 22530642

[B28] NINDS Exploratory Trials in Parkinson Disease (NET-PD) FS-ZONE Investigators. (2015). Pioglitazone in early Parkinson’s disease: a phase 2, multicentre, double-blind, randomised trial. Lancet Neurol. 14, 795–803. 10.1016/S1474-4422(15)00144-1 26116315 PMC4574625

[B29] Pagan F. Hebron M. Valadez E. H. Tores-Yaghi Y. Huang X. Mills R. R.. (2016). Nilotinib effects in Parkinson’s disease and dementia with Lewy bodies. J. Parkinsons Dis. 6, 503–517. 10.3233/JPD-160867 27434297 PMC5008228

[B30] Patrone C. Eriksson O. Lindholm D. (2014). Diabetes drugs and neurological disorders: new views and therapeutic possibilities. Lancet Diabetes Endocrinol. 2, 256–262. 10.1016/s2213-8587(13)70125-6 24622756

[B31] Pinilla-Ibarz J. Sweet K. Emole J. Fradley M. (2015). Long-term BCR-ABL1 tyrosine kinase inhibitor therapy in chronic myeloid leukemia. Anticancer Res. 35, 6355–6364. 26637844

[B32] Porkka K. Koskenvesa P. Lundán T. Rimpiläinen J. Mustjoki S. Smykla R.. (2008). Dasatinib crosses the blood-brain barrier and is an efficient therapy for central nervous system Philadelphia chromosome-positive leukemia. Blood 112, 1005–1012. 10.1182/blood-2008-02-140665 18477770

[B34] Putkonen N. Kukkonen J. P. Mudo G. Putula J. Belluardo N. Lindholm D.. (2011). Involvement of cyclin-dependent kinase-5 in the kainic acid-mediated degeneration of glutamatergic synapses in the rat hippocampus. Eur. J. Neurosci. 34, 1212–1221. 10.1111/j.1460-9568.2011.07858.x 21978141

[B35] Quintás-Cardama A. Cortes J. (2009). Molecular biology of bcr-abl1-positive chronic myeloid leukemia. Blood 113, 1619–1630. 10.1182/blood-2008-03-144790 18827185 PMC3952549

[B36] Remsing Rix L. L. Rix U. Colinge J. Hantschel O. Bennett K. L. Stranzl T.. (2009). Global target profile of the kinase inhibitor bosutinib in primary chronic myeloid leukemia cells. Leukemia 23, 477–485. 10.1038/leu.2008.334 19039322

[B37] Schlatterer S. D. Acker C. M. Davies P. (2011). c-Abl in neurodegenerative disease. J. Mol. Neurosci. 45, 445–452. 10.1007/s12031-011-9588-1 21728062 PMC3329755

[B38] Shah N. P. Kasap C. Weier C. Balbas M. Nicoll J. M. Bleickardt E.. (2008). Transient potent BCR-ABL inhibition is sufficient to commit chronic myeloid leukemia cells irreversibly to apoptosis. Cancer Cell 14, 485–493. 10.1016/j.ccr.2008.11.001 19061839

[B39] Shin J. H. Ko H. S. Kang H. Lee Y. Lee Y. I. Pletinkova O.. (2011). PARIS (ZNF746) repression of PGC-1α contributes to neurodegeneration in Parkinson’s disease. Cell 144, 689–702. 10.1016/j.cell.2011.02.010 21376232 PMC3063894

[B40] Singleton A. B. Farrer M. Johnson J. Singleton A. Hague S. Kachergus J.. (2003). α-synuclein locus triplication causes Parkinson’s disease. Science 302:841. 10.1126/science.1090278 14593171

[B41] Smith P. D. Crocker S. J. Jackson-Lewis V. Jordan-Sciutto K. L. Hayley S. Mount M. P.. (2003). Cyclin-dependent kinase 5 is a mediator of dopaminergic neuron loss in a mouse model of Parkinson’s disease. Proc. Natl. Acad. Sci. U S A 100, 13650–13655. 10.1073/pnas.2232515100 14595022 PMC263868

[B42] Spillantini M. G. Goedert M. (2016). Synucleinopathies: past, present and future. Neuropathol. Appl. Neurobiol. 42, 3–5. 10.1111/nan.12311 26819143

[B43] Spillantini M. G. Schmidt M. L. Lee V. M. Trojanowski J. Q. Jakes R. Goedert M. (1997). α-synuclein in Lewy bodies. Nature 388, 839–840. 9278044 10.1038/42166

[B44] Tanabe A. Yamamura Y. Kasahara J. Morigaki R. Kaji R. Goto S. (2014). A novel tyrosine kinase inhibitor AMN107 (nilotinib) normalizes striatal motor behaviors in a mouse model of Parkinson’s disease. Front. Cell. Neurosci. 8:50. 10.3389/fncel.2014.00050 24600352 PMC3929858

[B45] Weisberg E. Manley P. Mestan J. Cowan-Jacob S. Ray A. Griffin J. D. (2006). AMN107 (nilotinib): a novel and selective inhibitor for BCR-ABL. Br. J. Cancer 94, 1765–1769. 10.1038/sj.bjc.6603170 16721371 PMC2361347

[B46] Wen Z. Shu Y. Gao C. Wang X. Qi G. Zhang P.. (2014). CDK5-mediated phosphorylation and autophagy of RKIP regulate neuronal death in Parkinson’s disease. Neurobiol. Aging 35, 2870–2880. 10.1016/j.neurobiolaging.2014.05.034 25104559

[B47] Winslow A. R. Rubinsztein D. C. (2011). The Parkinson disease protein α-synuclein inhibits autophagy. Autophagy 7, 429–431. 10.4161/auto.7.4.14393 21157184 PMC3127221

[B48] Wyse R. K. Brundin P. Sherer T. B. (2016). Nilotinib–differentiating the hope from the hype. J. Parkinsons Dis. 6, 519–522. 10.3233/JPD-160904 27434298 PMC5044778

[B49] Yamamura Y. Morigaki R. Kasahara J. Yokoyama H. Tanabe A. Okita S.. (2013). Dopamine signaling negatively regulates striatal phosphorylation of Cdk5 at tyrosine 15 in mice. Front. Cell. Neurosci. 7:12. 10.3389/fncel.2013.00012 23420105 PMC3572678

